# Genes flow by the channels of culture: the genetic imprint of matrilocality in Ngazidja, Comoros Islands

**DOI:** 10.1038/s41431-018-0154-y

**Published:** 2018-04-30

**Authors:** Stéphane Mazières, Pauline Oviedo, Célia Kamel, Pascal Bailly, Caroline Costedoat, Jacques Chiaroni

**Affiliations:** 1 Aix Marseille Univ, CNRS, EFS, ADES, Marseille, France; 2 Etablissement Français du Sang PACA Corse, Biologie des groupes sanguins, Marseille, France

## Abstract

Post-marital residence of spouses is one of the architects of population genetic structure. In the present study, we tested how the place of residence of males and females in Ngazidja, Comoros Islands, has unequally channeled, by dispersal among villages, the male and female genetic diversity. Using sequences of the hypervariable segment I of the mitochondrial DNA (mtDNA HVS-I) and six Y-chromosome microsatellites (Y-STRs), we measured the genetic variation and male-to-female effective number of migrants ratios based on *F*_ST_ values and revealed a genetic structure mostly driven by male gene flow across villages. This genetic feature illustrates the uxori-matrilocality inherited from the Bantu expansion, though one exception exists in Bandamadji whose historically documented military status implied patrilocality in this locality.

## Introduction

The Comoros Islands are an archipelago of four islands, Ngazidja (Great Comoros in the Shingazidja dialect), Nzduani (Anjouan in Shindzuani), Mwali (Mohéli in Shimwali), and Mahore (Mayotte in Shimaore), located at the northern end of the Mozambique Channel in the Indian Ocean. Historical, social, linguistic, and genetic studies showed that the Comorian population arose from the encounter of East African Bantu metallurgists with Islamic Middle Eastern traders and, to a lesser degree, with Austronesian people [1–5].

The social structure in Comoros Islands results from the interactions between Islam’s laws and ancestral rites from East Africa, with a mixed distribution of matrilineal and patrilineal traits in various domains of social organization: kinship, inheritance of property, succession to titles, mode of residence, and detention of authority [2, 6]. In the four islands, the post-marital residence is largely uxori-matri-local: the husband resides with his wife and the daughters marry in the place of residence of their mother [2, 6]. Under African costumes and Islam’s laws, men are allowed to practice polygyny [2]. However, in order to rise its social position, a man has to realize his “great marriage” with a woman from his own village. This specific union happens while he is able to assume the great expenses needed for this unique event and during his adult’s life. Before, he may or has to conclude other unions that are not necessarily with women from his own maternal village, called “little marriage”. Men are thus considered as “itinerant” husbands moving from one to other village and will have between two to five unions in their life. Meanwhile, for her wedding the bride is given a house from her mother’s family. The oldest maternal uncle looks after her education and the one of her children, but these rules are not applied if the woman leaves the village. Furthermore, each Comorian individual and its social rights are as a priority identified by his matriclan, called *hinya*. All these conditions create a real socio-economic pressure to stay in the birth village for both men and women [2, 6].

Previous surveys in matrilocal and patrilocal structured populations have shown a contrasted pattern of within and between-population genetic variation when examined for paternally and maternally inherited genetic markers [7, 8]. Herein, we explored the uniparental genetic variation between five villages of the Great Comoros Island, Ngazidja, and tested to what extent the “little marriages” strengthened by the matrilocal and matrilineal social organization has left footprints in the genetic structure of this population.

## Material and methods

In accordance with French regulations of an ethical committee (Ministry of Research, record number DC-2008-164 and amendments, formally approved on December 15th, 2008), we collected with consent approval 86 male samples from five villages of Ngazidja: Bandamadji, Hahaya, Iconi, Male, and Mitsoudje. We included only unrelated men for at least two generations back in time and ensured the name of their birth village.

Blood collection and lab methods are detailed in ref. [9]. DNAs were sequenced for mtDNA HVS-I (GenBank accession numbers: MG878303-MG878379) and aligned to the Cambridge Reference Sequence [10], then screened for Y-STRs with the AmpFLSTR™ Yfiler™ PCR Amplification Kit (Applied Biosystem). Six of them (DYS389I, DYS389II, DYS390, DYS391, DYS392, and DYS393) were retained given their usefulness in genetic diversity estimates [11] (Table S1).

We first estimated the within-village genetic diversity and pairwise-difference (Pw), then tested the inter-village genetic structure with a measure of *F*_ST_ and *R*_ST_ genetic distances and distribution of the molecular variance (AMOVA) using the ARLEQUIN software [12]. We also performed two median joining networks using the program NETWORK 4.6.1.1 (Fluxus-Engineering) where we considered the HVS-I poly-C region according to ref. [13] and weighted the Y-STRs loci as a function of their variance [14] after exclusion of DYS389I-II as recommended. Lastly, we measured *N*_male_*m*_male_/*N*_female_*m*_*female*_ from *F*_ST_ values, the ratio between effective numbers of male and female migrants per generation, as described in ref. [15]. In this approach based on an island model, populations are stable, mutation is negligible and allele frequencies are assumed to result mainly from differences in migration rate per generation (*m*) and/or effective population size (N). Hence, the expected value of genetic differences between populations, *F*_ST_, could be written were *F*_ST_ = 1/(1 + *Nm*) for haploid systems [15].

## Results

We measured the female and male genetic diversity indices in the five Comoros villages understudy (Table 1). The main pattern is a higher male than female genetic diversity as observed in Hayaya, Male, and Mistoudje. Exceptions are Iconi which shows similar values for both uniparental markers, and overall Bandamadji which distinguishes with a higher mtDNA than Y-STRs genetic diversity.

**Table 1:** Summary statistics of mtDNA and Y-STRs genetic variation within and between five villages of Ngazidja

Genetic marker	Estimator	Bandamadji	Hahaya	Iconi	Male	Mitsoudje	Ngazidja
	Number of inhabitants	2000	2600	8000	13,000	5000	
mtDNA	Sample size	11	20	17	18	11	77
	Genetic diversity	0.946	0.742	0.985	0.699	0.927	0.864
	Pw	10.1	4.3	10.8	10.4	10.8	
	Mean *F*_ST_ (*p*-value) with the other villages	0.108 (0.205)	0.220 (0.000)	0.071 (0.164)	0.150 (0.016)	0.098 (0.088)	
	PS	0.73	0.20	0.76	0.33	0.55	
	C	1.22	2.50	1.13	2.25	1.38	
	% of variation among population						15.32 (0.000)
	% of variation within population						84.68 (0.000)
Y-STRs	Sample size	9	16	22	22	17	86
	Genetic diversity	0.722	0.942	0.952	0.987	0.971	0.953
	Pw	2.3	3.3	3.7	3.6	3.5	
	Mean *R*_ST_ (*p*-value) with the other villages	0.114 (0.036)	0.043 (0.110)	0.018 (0.279)	0.062 (0.216)	0.032 (0.340)	
	PS	0.44	0.31	0.68	0.73	0.71	
	C	1.80	1.60	1.29	1.16	1.21	
	% of variation among population						4.74 (0.000)
	% of variation within population						95.26 (0.000)
	Mean male-to-female number of migrants ratio	0.9	7.1	7.0	2.6	3.3	3.6

Values in the Ngazidja column were obtained from the AMOVA. Male-to-female number of migrants ratio was inferred from [15]

*Pw* mean number of pairwise differences, *PS* proportion of haplotypes observed only once in the population, *C* mean number of individuals carrying the same haplotype

**Fig. 1:**
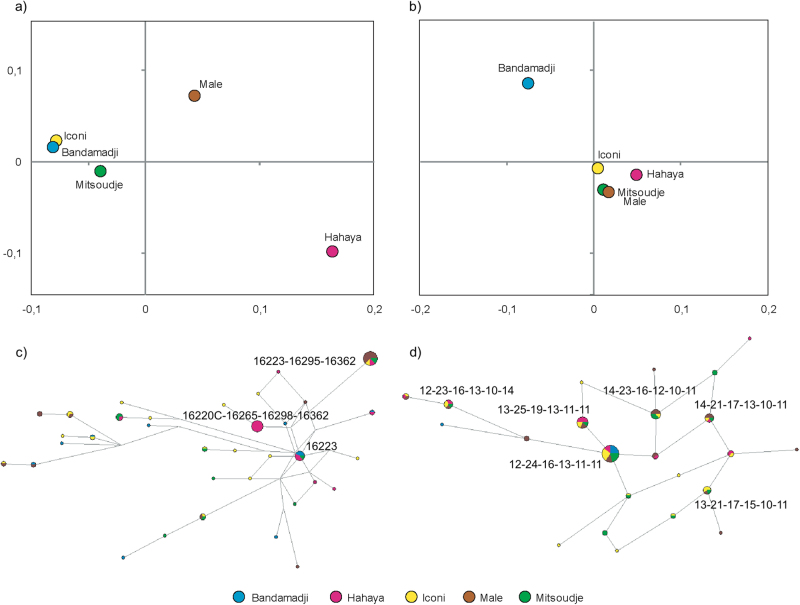
Multidimensional Scaling of *F*_ST_ for mtDNA HVS-I (**a**) and *R*_ST_ for Y-STRs (**b**) genetic distances and networks (NETWORK 5.0) of mtDNA HVS-I (**c**) and Y-STRs (**d**) haplotypes

We then depicted the *F*_ST_ and *R*_ST_ genetic distances onto a Multi Dimensional Scaling (MDS, Fig. 1a, b). For HVS-I, genetic similarities link the Iconi with Bandamadji while Hayaya and Male stand at peripheral positions. As far as the Y-STRs genetic variation is concerned, the Bandamadji village departs strongly from the four remaining villages, separated with non-significant *R*_ST_ values. When compared on a haplotype basis (Fig. 1c, d), networks mirror the genetic diversity pattern, as shown in Table 1. Iconi, Mitsoudje, and Bandamadji encapsulate the highest HVS-I haplotype diversity with numerous branches, while most of the Hayaya and Male mtDNAs are included into one main profile. As far as Y-STRs are concerned, every village is spread over the nodes, but Bandamadji which is represented by almost exclusively one profile. Networks also depict higher pairwise differences (Pw) for HVS-I than for Y-STRs (mean Pw_HVS-I_ = 9.3, mean Pw_YSTRs_ = 3.3, Mann–Whitney U-test, *p*-value = 0.037). Finally, one can notice twice more village-specific haplotypes for HVS-I (*n* = 24) than for Y-STRs (*n* = 12).

AMOVA points out that HVS-I presents 3.2-times more genetic variation amongst villages than Y-STRs (Table 1, respectively 15.32% and 4.74%, *p*-value = 0.000), but less within-village variation (ratio of 0.9, respectively 84.68% and 95.26%, *p*-value = 0.000). Due to the distinct genetic pattern of Bandamadji above-mentioned, we ran an additional AMOVA excluding this village. We noticed a strong increase of the contrasting patterns of within-population and between-population genetic variation as seen from HVS-I and Y-STRs, since the female genetic variation among populations reached almost 10-fold the male one (17.08% and 1.74%, *p*-value = 0.000).

**Fig. 2:**
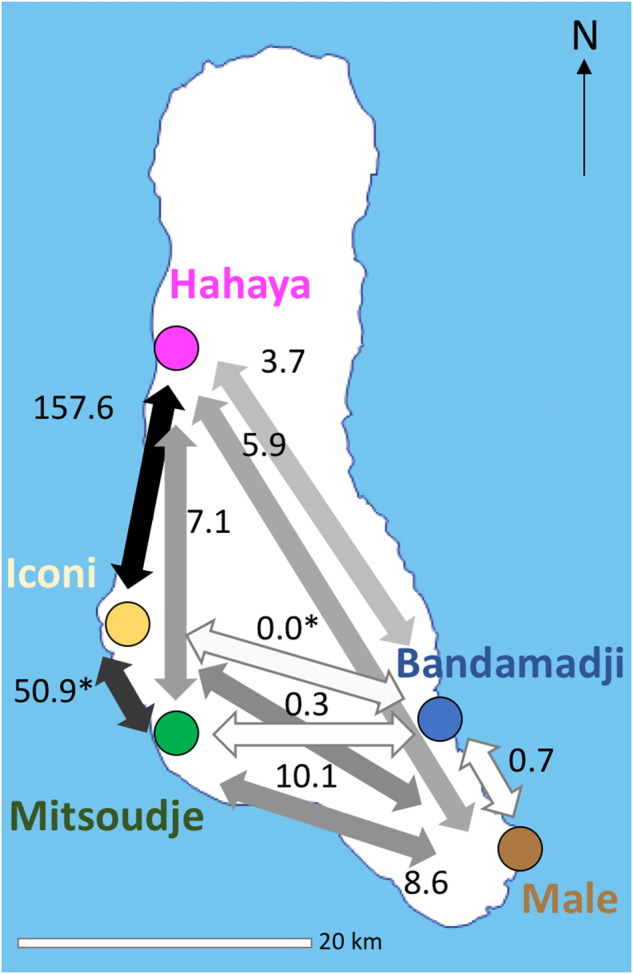
Intensity of male-to-female number of migrants ratio amongst the five villages understudy. Gray scale is proportional to log_10_(*F*_ST’_ ratio) from light (ratio<1, i.e., male<female) to dark (ratio>1, i.e., male>female). *Drawn from negative *F*_ST_ which were considered as 0.001. Background map was taken from www.d-maps.com©

Lastly, from the *F*_ST_ values we inferred the magnitude of male-to-female number of migrants ratio per generation for each village and across villages (Table 1 and Fig. 2). The main pattern in the island is a ratio above 1 (mean ratio = 3.5) where it ranges from 2.6 and 7.1 in four villages: Hayaya, Iconi, Male, and Mitsoudje. Exception is Bandamadji with a ratio of 0.9. Geographical distances do not account for the male and female genetic dissimilarities (Mantel test: km vs. R_*ST*YSTR_: *r* = −0.071, *p*-value = 0.533, km vs. F_*ST*mtDNA_: *r* = 0.5, *p*-value = 0.124).

## Discussion

A great variety of human social organizations defined by rules of marriage, residence, descent and mode of subsistence has been described worldwide [16], which in return can substantially impact the genetic diversity [17]. At the global scale, populations would be genetically more structured for paternally than for maternally inherited markers mirroring migrations of women in her mate’s residence and thus patrilocality [8]. Nevertheless, matrilocality rules other societies [18] and in the Indian Ocean, the earliest inhabitants of the Comoros originated from East Africa, as the extension of the matrilineal belt that encompassed Africa Bantu which opens to the east since the 11th century [3].

Uniparental genetic markers in Ngazidja indicate a male-oriented repartition of genetic diversity within and amongst villages, which is in accordance with described matrilocality. However, one exception is Bandamadji which behaves as a patrilocal population. Indeed, oral and historical knowledge evokes that Bandamadji was the military basis of one of the great Comorian clan M’Dombozi, where men have sworn fidelity to their sultan Hachim ben Ahmed (died 1889), and hence, had to stay to protect the city ([19] and Kassim Papa, pers. com.).

Finally, the Comorian villages are organized according to the social space in which males and females participate distinctly [2], and the present data agree with these ethno-historical records. The present study evidences that amongst and within human populations, genes also flow through the channels dug by culture and social organization. It especially illustrates the positive collaboration between geneticists and anthropologists to search together how distribution maps of biological phenomena and cultural phenomena shed light on each other [20].

## Electronic supplementary material

S1Table
