# Supplementary material for: Genes flow by the channels of culture: the genetic imprint of matrilocality in Ngazidja, Comoros Islands
Source: Eur J Hum Genet. 2018 Apr 30;26(8):1222–6. doi: 10.1038/s41431-018-0154-y (PMC6057983; doi:10.1038/s41431-018-0154-y)
Supplement: Supplementary file 1 — S1Table [file 41431_2018_154_MOESM1_ESM.docx]

**Table S1.** Y-STRs haplotypes collected in the five Comorian villages understudy. For each marker, reference sequence, starting nucleotide and repeat structure are given

| Village | haplotype | Number | dys389a | dys390 | dys389II | dys393 | dys391 | dys392 |
| --- | --- | --- | --- | --- | --- | --- | --- | --- |
|  |  |  | AC004617.3:g.62581  [TCTG]3[TCTA]r[9-17] | AC011289.4:g.69363  [TCTG]n [TCTA]m[TCTG]p[TCTA]q[17-28] | AC004617.3:g.62677  [TCTG]n[TCTA]p[TCTG]q [TCTA]r[24-34] | AC006152.4:g.21115  AGAT[ 9-17] | AC011302.3:g.30577  TCTA[6-14] | AC011745.4:g.52196  TAT[6-17] |
| Bandamadji | NBJ1 | 1 | 13 | 26 | 29 | 13 | 11 | 13 |
| Bandamadji | NBJ2 | 1 | 12 | 24 | 27 | 15 | 10 | 11 |
| Bandamadji | NBJ3 | 1 | 12 | 24 | 28 | 13 | 11 | 11 |
| Bandamadji | NBJ4 | 1 | 13 | 24 | 31 | 13 | 10 | 11 |
| Bandamadji | NBJ5 | 1 | 12 | 24 | 28 | 13 | 11 | 11 |
| Bandamadji | NBJ6 | 1 | 13 | 24 | 27 | 15 | 11 | 11 |
| Bandamadji | NBJ7 | 1 | 12 | 24 | 28 | 13 | 11 | 11 |
| Bandamadji | NBJ8 | 1 | 12 | 24 | 28 | 13 | 11 | 11 |
| Bandamadji | NBJ9 | 1 | 12 | 24 | 28 | 13 | 11 | 11 |
| Hahaya | NH1 | 1 | 12 | 21 | 29 | 12 | 10 | 13 |
| Hahaya | NH2 | 1 | 12 | 24 | 28 | 13 | 11 | 11 |
| Hahaya | NH3 | 1 | 13 | 21 | 31 | 14 | 10 | 11 |
| Hahaya | NH4 | 1 | 13 | 21 | 31 | 14 | 10 | 11 |
| Hahaya | NH5 | 1 | 13 | 21 | 31 | 13 | 10 | 11 |
| Hahaya | NH6 | 1 | 12 | 23 | 28 | 13 | 10 | 11 |
| Hahaya | NH7 | 1 | 12 | 24 | 28 | 13 | 11 | 11 |
| Hahaya | NH8 | 1 | 12 | 24 | 28 | 13 | 11 | 11 |
| Hahaya | NH9 | 1 | 12 | 23 | 29 | 13 | 11 | 15 |
| Hahaya | NH10 | 1 | 12 | 23 | 28 | 13 | 10 | 14 |
| Hahaya | NH11 | 1 | 12 | 23 | 28 | 13 | 10 | 14 |
| Hahaya | NH12 | 1 | 12 | 25 | 30 | 13 | 11 | 11 |
| Hahaya | NH13 | 1 | 12 | 25 | 30 | 13 | 11 | 11 |
| Hahaya | NH14 | 1 | 13 | 25 | 32 | 13 | 11 | 11 |
| Hahaya | NH15 | 1 | 13 | 25 | 32 | 13 | 11 | 11 |

*Table S1. Continued*

| Village | haplotype | Number | dys389a | dys390 | dys389II | dys393 | dys391 | dys392 |
| --- | --- | --- | --- | --- | --- | --- | --- | --- |
|  |  |  | AC004617.3:g.62581  [TCTG]3[TCTA]r[9-17] | AC011289.4:g.69363  [TCTG]n [TCTA]m[TCTG]p[TCTA]q[17-28] | AC004617.3:g.62677  [TCTG]n[TCTA]p[TCTG]q [TCTA]r[24-34] | AC006152.4:g.21115  AGAT[ 9-17] | AC011302.3:g.30577  TCTA[6-14] | AC011745.4:g.52196  TAT[6-17] |
| Hahaya | NH16 | 1 | 13 | 25 | 31 | 13 | 11 | 11 |
| Iconi | NI1 | 1 | 12 | 21 | 29 | 13 | 10 | 11 |
| Iconi | NI2 | 1 | 13 | 21 | 31 | 15 | 10 | 11 |
| Iconi | NI3 | 1 | 13 | 21 | 30 | 15 | 10 | 11 |
| Iconi | NI4 | 1 | 12 | 21 | 29 | 14 | 11 | 11 |
| Iconi | NI5 | 1 | 11 | 24 | 27 | 13 | 11 | 11 |
| Iconi | NI6 | 1 | 12 | 24 | 28 | 13 | 11 | 11 |
| Iconi | NI7 | 1 | 12 | 24 | 28 | 13 | 11 | 11 |
| Iconi | NI8 | 1 | 12 | 25 | 28 | 13 | 11 | 11 |
| Iconi | NI9 | 1 | 13 | 21 | 30 | 15 | 10 | 11 |
| Iconi | NI10 | 1 | 12 | 25 | 28 | 14 | 12 | 11 |
| Iconi | NI11 | 1 | 12 | 24 | 28 | 13 | 11 | 11 |
| Iconi | NI12 | 1 | 10 | 24 | 27 | 14 | 10 | 11 |
| Iconi | NI13 | 1 | 12 | 24 | 28 | 13 | 11 | 11 |
| Iconi | NI14 | 1 | 12 | 24 | 28 | 13 | 11 | 11 |
| Iconi | NI15 | 1 | 13 | 21 | 29 | 15 | 10 | 10 |
| Iconi | NI16 | 1 | 13 | 25 | 30 | 12 | 10 | 11 |
| Iconi | NI17 | 1 | 13 | 23 | 30 | 12 | 10 | 11 |
| Iconi | NI18 | 1 | 12 | 23 | 28 | 13 | 10 | 14 |
| Iconi | NI19 | 1 | 12 | 23 | 29 | 13 | 10 | 15 |
| Iconi | NI20 | 1 | 13 | 23 | 28 | 15 | 10 | 10 |
| Iconi | NI21 | 1 | 13 | 25 | 31 | 13 | 11 | 11 |
| Iconi | NI22 | 1 | 13 | 23 | 28 | 13 | 10 | 14 |
| Male | NM1 | 1 | 13 | 24 | 29 | 13 | 11 | 13 |
| Male | NM2 | 1 | 13 | 24 | 29 | 13 | 11 | 13 |

Table S1. Continued

| Village | haplotype | Number | dys389a | dys390 | dys389II | dys393 | dys391 | dys392 |
| --- | --- | --- | --- | --- | --- | --- | --- | --- |
|  |  |  | AC004617.3:g.62581  [TCTG]3[TCTA]r[9-17] | AC011289.4:g.69363  [TCTG]n [TCTA]m[TCTG]p[TCTA]q[17-28] | AC004617.3:g.62677  [TCTG]n[TCTA]p[TCTG]q [TCTA]r[24-34] | AC006152.4:g.21115  AGAT[ 9-17] | AC011302.3:g.30577  TCTA[6-14] | AC011745.4:g.52196  TAT[6-17] |
| Male | NM3 | 1 | 13 | 25 | 31 | 13 | 10 | 11 |
| Male | NM4 | 1 | 13 | 21 | 30 | 14 | 10 | 9 |
| Male | NM5 | 1 | 13 | 20 | 31 | 15 | 10 | 11 |
| Male | NM6 | 1 | 13 | 21 | 31 | 13 | 11 | 11 |
| Male | NM7 | 1 | 14 | 21 | 31 | 13 | 10 | 11 |
| Male | NM8 | 1 | 14 | 21 | 31 | 13 | 10 | 11 |
| Male | NM9 | 1 | 13 | 21 | 30 | 15 | 10 | 11 |
| Male | NM10 | 1 | 13 | 23 | 31 | 12 | 10 | 11 |
| Male | NM11 | 1 | 13 | 23 | 28 | 12 | 12 | 11 |
| Male | NM12 | 1 | 12 | 23 | 28 | 13 | 10 | 14 |
| Male | NM13 | 1 | 12 | 25 | 28 | 13 | 11 | 11 |
| Male | NM14 | 1 | 12 | 24 | 28 | 13 | 10 | 11 |
| Male | NM15 | 1 | 13 | 24 | 30 | 13 | 10 | 11 |
| Male | NM16 | 1 | 15 | 23 | 29 | 13 | 10 | 11 |
| Male | NM17 | 1 | 13 | 23 | 28 | 12 | 10 | 11 |
| Male | NM18 | 1 | 12 | 23 | 29 | 13 | 11 | 15 |
| Male | NM19 | 1 | 13 | 24 | 31 | 13 | 11 | 11 |
| Male | NM20 | 1 | 13 | 23 | 28 | 12 | 10 | 11 |
| Male | NM21 | 1 | 13 | 23 | 32 | 13 | 10 | 11 |
| Male | NM22 | 1 | 14 | 23 | 28 | 12 | 10 | 11 |
| Mitsoudje | NMD1 | 1 | 12 | 21 | 29 | 12 | 11 | 11 |
| Mitsoudje | NMD2 | 1 | 13 | 22 | 30 | 14 | 10 | 11 |
| Mitsoudje | NMD3 | 1 | 14 | 21 | 31 | 15 | 10 | 11 |
| Mitsoudje | NMD4 | 1 | 12 | 21 | 30 | 13 | 10 | 11 |
| Mitsoudje | NMD5 | 1 | 12 | 24 | 28 | 13 | 11 | 11 |

*Table S1. Continued*

| Village | haplotype | Number | dys389a | dys390 | dys389II | dys393 | dys391 | dys392 |
| --- | --- | --- | --- | --- | --- | --- | --- | --- |
|  |  |  | AC004617.3:g.62581  [TCTG]3[TCTA]r[9-17] | AC011289.4:g.69363  [TCTG]n [TCTA]m[TCTG]p[TCTA]q[17-28] | AC004617.3:g.62677  [TCTG]n[TCTA]p[TCTG]q [TCTA]r[24-34] | AC006152.4:g.21115  AGAT[ 9-17] | AC011302.3:g.30577  TCTA[6-14] | AC011745.4:g.52196  TAT[6-17] |
| Mitsoudje | NMD6 | 1 | 12 | 24 | 28 | 13 | 11 | 11 |
| Mitsoudje | NMD7 | 1 | 12 | 24 | 28 | 13 | 11 | 11 |
| Mitsoudje | NMD8 | 1 | 10 | 24 | 27 | 14 | 10 | 11 |
| Mitsoudje | NMD9 | 1 | 11 | 24 | 27 | 13 | 11 | 11 |
| Mitsoudje | NMD10 | 1 | 12 | 24 | 28 | 13 | 10 | 11 |
| Mitsoudje | NMD11 | 1 | 13 | 21 | 29 | 15 | 10 | 10 |
| Mitsoudje | NMD12 | 1 | 13 | 21 | 29 | 12 | 10 | 11 |
| Mitsoudje | NMD13 | 1 | 12 | 23 | 28 | 13 | 10 | 14 |
| Mitsoudje | NMD14 | 1 | 13 | 25 | 31 | 13 | 11 | 11 |
| Mitsoudje | NMD15 | 1 | 14 | 23 | 30 | 12 | 10 | 11 |
| Mitsoudje | NMD16 | 1 | 14 | 23 | 30 | 12 | 10 | 11 |
| Mitsoudje | NMD17 | 1 | 13 | 23 | 30 | 12 | 10 | 11 |
